# The value of indocyanine green clearance assessment to predict postoperative liver dysfunction in patients undergoing liver resection

**DOI:** 10.1038/s41598-019-44815-x

**Published:** 2019-06-10

**Authors:** Christoph Schwarz, Immanuel Plass, Fabian Fitschek, Antonia Punzengruber, Martina Mittlböck, Stephanie Kampf, Ulrika Asenbaum, Patrick Starlinger, Stefan Stremitzer, Martin Bodingbauer, Klaus Kaczirek

**Affiliations:** 1 Department of Surgery, Division of General Surgery, Medical University Vienna, Vienna, Austria; 2 Section for Medical Statistics, Medical University Vienna, Vienna, Austria; 3 Department of Biomedical Imaging and Image Guided Therapy, Medical University Vienna, Vienna, Austria

**Keywords:** Risk factors, Gastroenterology

## Abstract

Postoperative liver dysfunction remains a major concern following hepatic resection. In order to identify patients who are at risk of developing liver dysfunction, indocyanine green (ICG) clearance has been proposed to predict postoperative liver function. All patients who underwent liver resection at the Medical University Vienna, Austria between 2006 and 2015 with preoperative ICG clearance testing (PDR, R15) were analyzed in this study. Postoperative liver dysfunction was analyzed as defined by the International Study Group of Liver Surgery. Overall, 698 patients (male: 394 (56.4%); female: 304 (43.6%)) with a mean age of 61.3 years (SD: 12.9) were included in this study, including 313 minor liver resections (44.8%) and 385 major liver resections (55.2%). One hundred and seven patients developed postoperative liver dysfunction after liver resection (15.3%). Factors associated with liver dysfunction were: male sex (p = 0.043), major liver resection (p < 0.0001), and preoperative ICG clearance (PDR (p = 0.002) and R15 (p < 0.0001)). Notably ICG clearance was significantly associated with liver dysfunction in minor and major liver resections respectively and remained a predictor upon multivariable analysis. An optimal cut-off for preoperative ICG clearance to accurately predict liver dysfunction was PDR < 19.5%/min and R15 > 5.6%. To the best of our knowledge, this is the largest study analyzing the predictive value of preoperative ICG clearance assessment in patients undergoing liver resection. ICG clearance is useful to identify patients at risk of postoperative liver dysfunction.

## Introduction

Liver resection has become the treatment of choice for a wide range of benign and malignant disease entities. While severe morbidity and mortality has decreased in specialized centers to 20 and 1–3% respectively^1–3^, postoperative liver dysfunction still remains a major concern associated with a significant incidence of liver-related deaths^4,5^. Even in healthy patients without underlying liver damage, the incidence of hepatic dysfunction following major liver resection is estimated to be approximately 5%^6^. Known risk factors are the extent of resection, intraoperative blood loss, preoperative chemotherapy but most importantly preoperative liver function^7–9^. In patients with impaired liver function even a small resection can result in a fatal outcome^3,10^. Thus, a proper patient selection is crucial to exclude patients that may not benefit from hepatectomy.

In order to reduce mortality due to postoperative liver dysfunction, several strategies have been established to identify patients at risk. Indocyanine green (ICG) clearance has been validated as a valuable tool for identifying patients with impaired preoperative liver function^11,12^. In liver resection ICG clearance has been proposed to define patients, who are at risk of developing postoperative liver dysfunction^13,14^ or surgical complications^15^.

Currently, data on the value of preoperative ICG clearance testing to predict the development of postoperative liver dysfunction are scarce and studies are limited to small patient numbers. Moreover, there exists no analysis investigating the predictive value of ICG clearance with respect to the extent of liver resections (minor versus major liver resection). Therefore, we performed an extensive analysis of all patients who underwent liver resection in our center with available preoperative ICG clearance testing results and determined the predictive value regarding postoperative liver dysfunction.

## Methods

This is a retrospective study investigating the predictive value of ICG clearance testing with respect to postoperative outcome after liver resection. All patients with available preoperative ICG clearance testing results who underwent elective liver resection of at least one segment between January 2005 and December 2016 were included in this analysis. Patients who underwent non-anatomical resections were excluded from this analysis. Overall 1008 patients underwent hepatic resection within the study period and preoperative ICG clearance testing results were available in 698 patients (63.2%). The type of liver resection was defined as major and minor resections according to the IHPBA Brisbane 2000 nomenclature (≤2 segments: minor; >2 segments: major)^16^. The study was reviewed and approved by the institutional review board of the Medical University Vienna. All methods were performed in accordance with the relevant guidelines and regulations. Due to the retrospective nature of this study the IRB waived the need to obtain informed consent.

### ICG clearance testing

ICG clearance was measured as previously described^15^. In brief, patients received 0.25 mg/kg ICG intravenously on the day before the liver resection. The plasma disappearance rate (PDR) and the ICG retention rate (R15) were measured by pulse spectrometry with a LiMON device (Pulsion Medical Systems, Munich, Germany). The surgical strategy was not changed upon the results from the ICG clearance testing.

### Study endpoints

The primary endpoint was the incidence of liver dysfunction defined by the ISGLS criteria as abnormal bilirubin levels and prothrombin time on or after postoperative day five^17^. Secondary endpoints were postoperative complications (according to the Clavien-Dindo classification^18^, lengths of stay and overall survival.

### Statistics

Metric data were expressed as means with SD or median with interquartile range (Q1–Q3) and comparison between groups was performed with the Mann-Whitney U test or an unpaired t-test as indicated. Categorical values were compared with Fishers-exact test or a chi-square test. A logistic regression was used to model occurrence of liver dysfunction. Results are presented with odds ratios (OR) and corresponding 95% confidence intervals (95% CI). For this IGG clearance were log-transformed with a basis of 10. Survival probabilities were calculated using a Kaplan-Meier-analysis and group comparison was performed using a log-rank test. Al performed tests are based on a significance level of 0.05. Statistical analysis was performed using GraphPad Prism, version 6 (GraphPad Prism Software®, La Jolla, CA) and SAS (©SAS Institute Inc., Cary, NC, USA).

## Results

### Patient characteristics

Patient characteristics are show in Table 1. Overall, 698 patients were included in this study. The majority of patients were male (n = 394; 56.4%) and underwent major liver resection (n = 385; 55.2%). The indications for liver resections were predominantly metastases (n = 390; 55.9%) followed by primary liver cancer (31.2%) and benign diseases (12.9%). The median follow-up was 23.3 months (8.9–49.7).

**Table 1:** Patient characteristics.

	Overall, n = 698	Minor LR, n = 313	Major LR, n = 385	p-value
Sex [male], n (%)	394 (56.4)	187 (59.7)	207 (53.8)	p = 0.125
Age [years], mean (SD)	61.3 (12.9)	61.5 (12.8)	61.1 (13.1)	p = 0.648
BMI [kg/m2], mean (SD)	26.8 (11.4)	27.6 (16.3)	26.1 (4.4)	p = 0.101
Indication for LR, n (%)				
Benign	90 (12.9)	46 (14.7)	44 (11.4)	p = 0.213
Metastasis	390 (55.9)	185 (59.1)	205 (53.2)	p = 0.126
Primary liver tumor	218 (31.2)	82 (26.2)	136 (35.3)	p = 0.011*
Comorbidities, n (%)				
Coronary heart disease	41 (5.9)	23 (7.3)	18 (4.7)	p = 0.147
IDDM	24 (3.4)	9 (2.9)	15 (3.9)	p = 0.535
NIDDM	67 (9.6)	35 (11.2)	32 (8.3)	p = 0.245
Obesity	106 (15.2)	53 (16.2)	53 (13.8)	p = 0.289
Arterial hypertension	215 (30.8)	93 (29.7)	122 (31.7)	p = 0.621
No comorbidities	206	88 (28.1)	118 (30.6)	p = 0.505
Portal vein embolization, n (%)	63 (9)	3 (1)	60 (15.6)	p < 0.0001****
First LR, n (%)	612 (87.7)	267 (85.3)	345 (89.6)	p = 0.105
Repeat LR, n (%)	86 (12.3)	46 (14.7)	40 (10.4)	p = 0.105
Type of LR, n (%)				p = 0.0003***
Open LR, n (%)	678 (97.1)	296 (94.6)	382 (99.2)	
Laparoscopic LR, n (%)	20 (2.9)	17 (5.4)	3 (0.8)	
Total vascular exclusion, n (%)	22 (3.2)	4 (1.3)	18 (4.7)	p = 0.015*
Grade of fibrosis, n (%)				p = 0.292
0	231 (33.1)	104 (33.2)	127 (33)	
I	252 (36.1)	110 (35.1)	142 (36.9)	
II	55 (7.9)	27 (8.6)	28 (7.3)	
III	14 (2)	6 (1.9)	8 (2.1)	
IV	45 (6.4)	27 (8.6)	18 (4.7)	
ICG clearance				
PDR, median (Q1–Q3)	21.1 (17.7–25.3)	21.4 (18–25.7)	17.4 (21–25.1)	p = 0.448
R15, median (Q1–Q3)	4 (2–7)	4 (2–7)	2 (4–7)	p = 0.776

*p ≤ 0.05; ***p ≤ 0.001; ****p ≤ 0.0001.

### ICG clearance

The median PDR was 21.1%/min (17.7–25.3) and the median R15 was 4.0% (2.0–7.0). There was no significant difference between patients undergoing major or minor resections with respect to preoperative ICG clearance testing. However, patients with hepatocellular carcinoma (HCC) had a significantly impaired ICG clearance compared to patients with other indications for liver resection (metastasis, cholangiocarcinoma or benign disease) (PDR: 19.5%/min (16.4–25) vs. 21.6%/min (18–25.7); p = 0.009) (Suppl. Figure 1a,b). Additionally, patients with HCC a significantly higher fibrosis score in the resected specimen **(**Suppl. Figure 1c**)**. Of note, there was no significant difference in PDR (p = 0.152) or R15 (p = 0.251) in patients with or without preoperative portal vein embolization (PVE). When analyzing the association between MELD score and ICG clearance we found a significant correlation between PDR (p < 0.0001; r = −0.236) and R15 (p < 0.0001; r = 0.238) **(**Suppl. Figure 2**)**.

### Postoperative liver dysfunction

**Figure 1:**
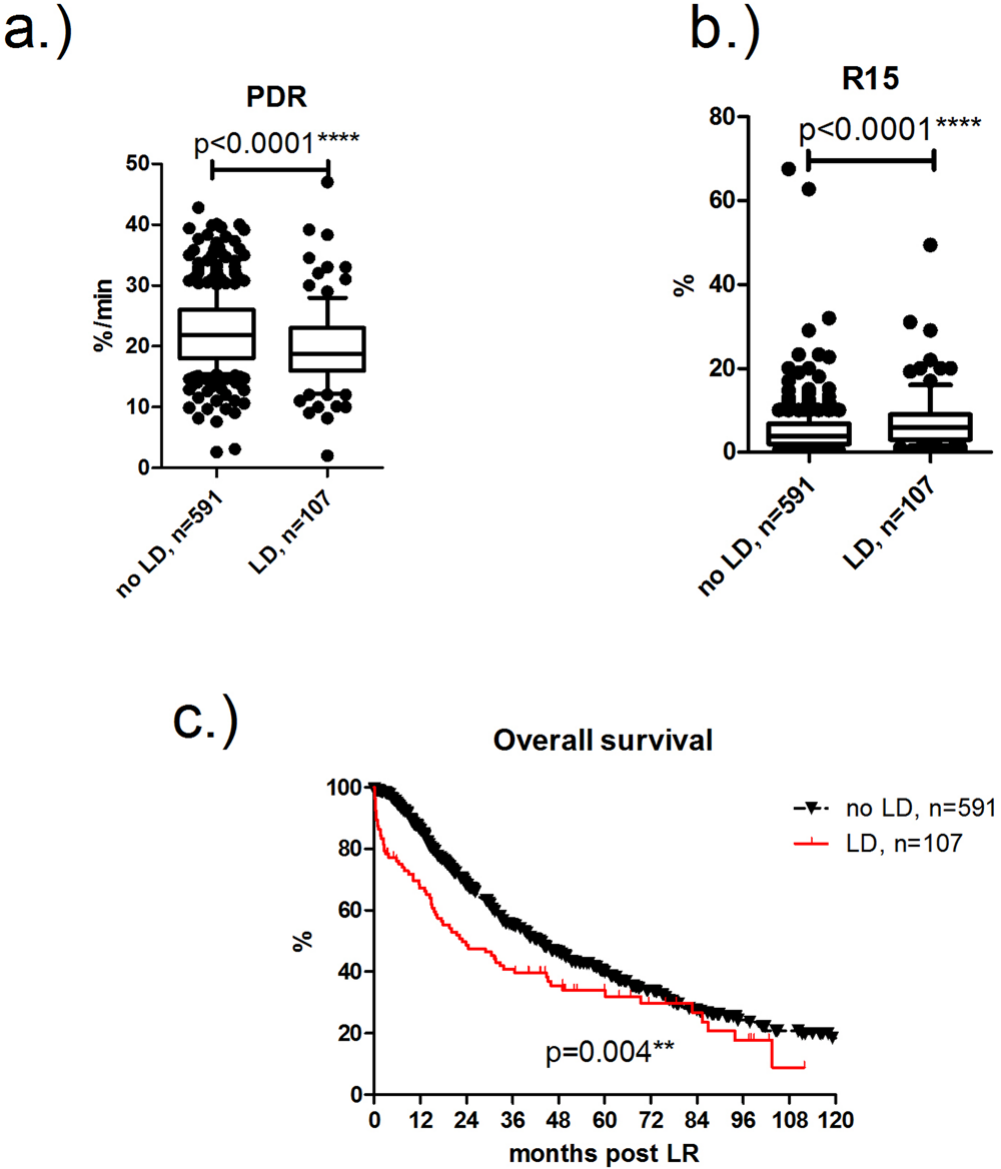
ICG clearance and liver dysfunction: Patients with postoperative liver dysfunction had significantly higher PDR (**a**) and R15 (**b**) levels. Overall survival was significantly diminished in patients with liver dysfunction compared to patients without (p = 0.004). **p ≤ 0.01; **** p ≤ 0.0001.

Overall, 107 patients developed postoperative liver dysfunction after liver resection (15.3%). In these patients, PDR levels were significantly lower compared to patients with normal postoperative liver function (18.8 (16.1–23.1) vs. 21.8 (18–26); p < 0.0001) (Fig. 1a). Conversely, R15 was significantly higher in patients with liver dysfunction (6 (3–8.9) vs. 3.9 (2–6.8); p < 0.0001) (Fig. 1b). Notably, patients with liver dysfunction had a significantly shorter median overall survival compared to patients without liver dysfunction (median 22.9 vs 43.1 months; OR 1.9 (1.4–2.5), p = 0.004) (Fig. 1c).

Factors associated with liver dysfunction in a univariate analysis were: male sex (p = 0.043), type of resection (major, minor liver resection) (p < 0.0001), and preoperative ICG clearance (PDR (p = 0.002) and R15 (p < 0.0001)). In a multivariate analysis male sex (OR 1.76 (95% CI 1.02–3.02); p = 0.043); major resection (OR 8.5 (95% CI 4.3–16.5); p < 0.0001) and a higher ICG clearance (R15, based on a log10 transformation) (OR 2.15 (95% CI 1.05–1.00) p = 0.035) remained with a significantly increased risk for liver dysfunction (Table 2).

**Table 2:** Predictors of liver dysfunction.

	Univariate Logistic Regression		Multivariable Logistic Regression	
	OR (95% CI)	p- value	OR (95% CI)	p- value
Age	1.01 (1.00–1,03)	0.135	0.99 (0.97–1.02)	0.629
Sex (male versus female)	**1**.**56 (1**.**01–2**.**40)**	**0**.043*	**1**.**76 (1**.**02–3**.**02)**	**0**.043*
First/Re LR (Re vs. first)	0.61 (0.30–1.26)	0.185	0.67 (0.29–1,55)	0.347
Type of resection (major vs. minor)	**6**.**67 (3**.**84–12**.**50)**	<**0**.0001****	**8**.**47 (4**.**26–16**.**95)**	<**0**.0001****
Primary liver tumor vs. liver metastasis	1.62 (1.05–2.50)	0.018	1.04 (0.60–1.81)	0.887
Comorbidities (yes vs. no)	1.03 (0.65–1.61)	0.894	0.85 (0.48–1.54)	0.587
Grade of fibrosis	**1**.**22 (1**.**02–1**.**46)**	**0**.030*	1.17 (0.93–1.46)	0.179
Portal vein embolization	**3**.**19 (1**.**80–5**.**65)**	<**0**.0001****	1.65 (0.84–3.24)	0.148
ICG PDR	**0**.**10 (0**.**03–0–42)**	**0**.002***		
ICG R15	**3**.**14 (1**.**78–5**.**54)**	<**0**.0001****	**2**.**15 (1**.**05–1**.**00)**	**0**.035*
Thrombocytes pre LR	**0**.**999 (0**.**996–1**.**001)**	0.272	0.995 (0.995–1.001)	0.225

*p ≤ 0.05; ***p ≤ 0.001; ****p ≤ 0.0001.

Of note, ICG clearance was significantly associated with liver dysfunction in minor and major liver resections, respectively.

### Optimal cut-off values for ICG clearance testing

The best cut-off value for predicting liver dysfunction was calculated by using the Youden-index. An optimal cut-off for preoperative ICG clearance to accurately predict liver dysfunction was a PDR < 19.5%/min and an R15 > 5.6%. Patients with impaired ICG clearance according to these cut-off values were significantly older, were more likely to be male and had less likely a benign cause for liver resection (Table 3). Notably, the grade of fibrosis in the liver was higher compared to patients who did not fulfill these criteria.

**Table 3:** Preoperative ICG clearance testing cut-off levels.

	PDR ≥ 19.5%/min, n = 429	PDR < 19.5%/min, n = 269	p-value	R15 > 5.6%, n = 439	R15 ≤ 5.6%, n = 249	p-value
Sex [male], n (%)	213 (49.7)	181 (67.3)	p < 0.0001****	221 (50.3)	167 (67.1)	p < 0.0001****
Age [years], mean (SD)	58.5 (13.1)	65.8 (11.4)	p < 0.0001****	58.6 (13.2)	66.2 (11.1)	p < 0.0001****
BMI [kg/m^2^], mean (SD)	27 (14.1)	26.5 (4.6)	p = 0.557	26.9 (13.9)	26.6 (4.6)	p = 0.726
Indication for LR, n (%)						
Benign	69 (16.1)	21 (7.8)	p = 0.007**	72 (16.4)	18 (7.2)	p = 0.0006***
Metastasis	231 (53.8)	159 (59.1)	p = 0.184	235 (53.5)	148 (59.4)	p = 0.151
Primary liver tumor	129 (30.1)	89 (33.1)	p = 0.403	132 (30.1)	83 (33.3)	p = 0.393
Comorbidities, n (%)						
Coronary heart disease	19 (4.4)	22 (8.2)	p = 0.047*	21 (4.8)	20 (8)	p = 0.095
IDDM	13 (3)	11 (4.1)	p = 0.524	14 (3.2)	9 (3.6)	p = 0.826
NIDDM	37 (8.6)	30 (11.2)	p = 0.292	39 (8.9)	28 (11.2)	p = 0.349
Obesity	68 (15.9)	38 (14.1)	p = 0.589	65 (14.8)	38 (15.3)	p = 0.912
Arterial hypertension	118 (27.5)	97 (36.1)	p = 0.019*	129 (29.4)	85 (34.1)	p = 0.200
No comorbidities	127 (29.6)	79 (29.4)	p = 1.000	128 (29.2)	75 (30.1)	p = 0.795
Portal vein embolization, n (%)	33 (7.7)	30 (11.2)	p = 0.136	36 (8.2)	27 (10.8)	p = 0.272
First LR, n (%)	380 (88.6)	232 (86.2)	p = 0.408	388 (88.4)	214 (85.9)	p = 0.401
Repeat LR, n (%)	49 (11.4)	37 (13.8)	p = 0.408	51 (11.6)	35 (14.1)	p = 0.401
Minor LR, n (%)	200 (46.6)	113 (42)	p = 0.242	200 (45.6)	109 (43.8)	p = 0.690
Major LR, n (%)	229 (53.4)	156 (58)	p = 0.242	239 (54.4)	140 (56.2)	p = 0.690
Type of LR, n (%)						
Open LR, n (%)	414 (96.5)	264 (98.1)	p = 0.249	424 (96.6)	244 (98)	p = 0.351
Laparoscopic LR, n (%)	15 (3.5)	5 (1.9)	p = 0.249	15 (3.4)	5 (2)	p = 0.351
Total vascular exclusion, n (%)	13 (3)	9 (3.3)	p = 0.827	14 (3.2)	7 (2.8)	p = 1.000
Grade of fibrosis, n (%)			p = 0.0049**			p = 0.002**
0	149 (34.7)	82 (30.5)		151 (34.4)	74 (29.7)	
I	161 (37.5)	91 (33.8)		165 (37.6)	85 (34.1)	
II	31 (7.2)	24 (8.9)		33 (7.5)	22 (8.8)	
III	6 (1.4)	8 (3)		6 (1.4)	8 (3.2)	
IV	16 (3.7)	29 (10.8)		15 (3.4)	29 (11.6)	
ICG clearance						
PDR, median (Q1–Q3)	24.5 (22–27.5)	17 (15–18)	p < 0.0001****	25 (21.5–27.4)	17 (15–18)	p < 0.0001****
R15, median (Q1–Q3)	2.5 (1.6–3.9)	8 (6.1–10.4)	p < 0.0001****	2.6 (1.6–3.9)	8 (7–10.5)	p < 0.0001****
Liver dysfunction, n (%)	45 (10.5)	62 (23)	p < 0.0001****	46 (10.5)	61 (24.5)	p < 0.0001****
Clavien-Dindo Grade, n (%)			p = 0.002**			p = 0.0005***
I	33 (7.7)	33 (12.3)		36 (8.2)	30 (12)	
II	55 (12.8)	48 (17.8)		55 (12.5)	47 (18.9)	
IIIa	37 (8.6)	21 (7.8)		38 (8.7)	18 (7.2)	
IIIb	38 (8.9)	30 (11.2)		38 (8.7)	29 (11.6)	
IVa	2 (0.5)	1 (0.4)		2 (0.5)	1 (0.4)	
IVb	0	2 (0.7)		0	2 (0.8)	
V	12 (2.8)	12 (4.5)		11 (2.5)	12 (4.8)	
Hospital stay duration, median (Q1–Q3) §	10.5 (8–15)	12 (8–18)	p < 0.0001****	10 (7–14)	12 (8–17)	p < 0.0001****

*p ≤ 0.05; **p ≤ 0.01; ***p ≤ 0.001; **** p ≤ 0.0001.

**Figure 2:**
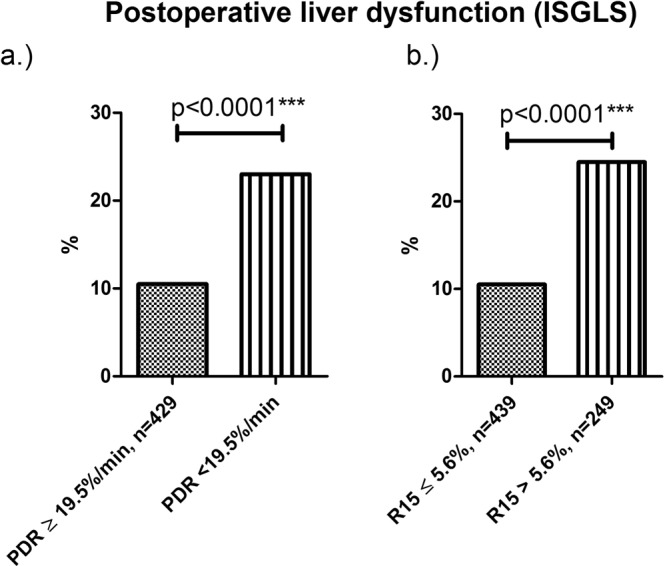
ICG clearance cut-off levels and liver dysfunction: By using a cut-off with a PDR < 19.5% min and an R15 of more than 5.6% allowed for defining a cohort of patients with a significantly increased risk of developing liver dysfunction. ***p ≤ 0.001.

Most strikingly, patients with impaired ICG clearance developed two times more likely a postoperative liver dysfunction (Fig. 2) that also resulted in more, and more severe complications and ultimately to a significantly prolonged length of hospitalization. Sensitivity for R15 (>5.6%) was 57% and the specifity was 66.5% resulting in a false positive (FP) rate of 33.5% and a false negative (FN) rate of 43%. For PDR (<19.5%/min) sensitivity was 57.4% and specifity 64.9% (FP: 35.1% FN: 42.5%).

## Discussion

To the best of our knowledge, this is the largest study analyzing the predictive value of preoperative ICG clearance assessment in patients undergoing liver resection with respect to postoperative liver dysfunction and clinical outcome. ICG clearance identified patients at risk of postoperative liver dysfunction and significantly worse clinical outcome. Especially in patients undergoing major liver resection, preoperative ICG clearance testing has proven to be clinically relevant given the relatively high incidence of liver dysfunction compared to minor liver resections. Short-term survival was significantly diminished in patients who developed liver dysfunction after liver resection, however long-term outcome was similar to patients without liver dysfunction. These findings are line with previous reports that post-operative complications don’t affect long-term outcome^19–21^. Ultimately, as shown by Padickakudy *et al*. it might be that serotonin plays a bivotal role in liver regeneration and tumor progression. While patients with high serotonin levels have a lower risk for developing post-operative liver dysfunction, the risk for developing tumor progression is significantly higher compared to patients with low levels of serotonin levels (who are vice versa at higher risk for developing liver dysfunction)^22^. However, these findings remain to be verified in future and larger clinical studies.

Several approaches are currently pursued to determine preoperative liver function including functional imaging-based analysis^23^, analysis of intra-platelet serotonin^24^ or HVPG measuring^25,26^. In contrast to most of these strategies, ICG clearance testing is cheap, non-invasive and readily available allowing for discrimination between high and low risk patients.

In this study, patients with a worse ICG clearance were generally older, more likely to be male and had a higher grade of liver fibrosis in the resected specimen compared to patients with normal values. These results are in line with several other studies showing a connection between ICG clearance and liver fibrosis^11,27^. However, in a previous publication Wong *et al*. reported no difference between R15 rates in patients with or without liver cirrhosis^28^. This discrepancy might be explained by the smaller patient number and the relatively high proportion of HCC patients compared to our study.

A worse ICG clearance was associated with the development of postoperative liver dysfunction. These results are in accordance with previous smaller studies^13,29,30^. By using a cut-off of a PDR of <19.5%/min and an R15 > 5.6% a group of patients was identified with a significantly worse outcome. Tomimaru *et al*. reported that platelet count was superior to ICG clearance testing (R15) in the prediction of postoperative liver dysfunction^31^. In the present study we didn’t find any association between platelet numbers and the incidence of liver dysfunction, which may be explained by the high proportion of patients with normal liver function.

In a recent study by Zou *et al*. R15 was significantly associated with liver dysfunction in minor liver resections but not major liver resections^32^. These results are contrary to our study as ICG clearance was found to be associated with liver dysfunction in both minor and major liver resections. We assume that the conflicting results can be explained by the relatively low incidence of liver dysfunction in minor liver resections in our cohort and by the lower percentage of HCC patients, which are known for a high percentage of liver damage^33^.

The clinical consequences of an impaired liver function need to be carefully considered when selecting patients for liver resection. On the one hand extensive resection should be avoided, parenchymal sparing surgery and combinations with intraoperative ablations of small lesions instead of major liver resections should be favored^34,35^. Alternative approaches to prevent postoperative liver dysfunction are the augmentation of the future liver remnant (FLR) by performing portal vein embolization or the ALPPS procedure. Consequently, the question which reference of FLR should be anticipated in patients with poor ICG clearance needs to be evaluated in future clinical studies.

We are aware that there are several limitations of this analysis which are inherent to the retrospective nature of this study. However, a large patient number and a long follow up may outweigh the latter restrictions. The large overlap of PDR and R15 values observed in patients with and without liver dysfunction certainly limits the predictive capacity of ICG clearance testing as a single tool to evaluate a patient before liver resection. Thus, we suggest to combine the results with clinical status, type of hepatectomy and other important factors including established liver damage, the use of neoadjuvant chemotherapy or any other relevant comorbidities to estimate the risk of liver resection.

We further narrowed the tested variables in the uni- and multivariate analysis to clinically relevant and already established factors that may contribute to liver dysfunction, thus, there is the chance that we missed further parameters which are connected to liver dysfunction. However, to prevent a model overfit and to have reproducible results we limited the tested values to the (in our opinion) most important variables.

## Conclusions

We conclude that in patients with poor ICG clearance at a cut-off of the PDR of 19.5%/min and an R15 of 5.6% in combination with other risk factors such as male sex, major liver resections should be considered with caution and patients informed accordingly. Besides that, ICG clearance testing is a valuable tool to identify patients at risk of developing postoperative liver dysfunction.

## Supplementary information

Supplementary figures
