# Supplementary material for: The value of indocyanine green clearance assessment to predict postoperative liver dysfunction in patients undergoing liver resection
Source: Sci Rep. 2019 Jun 10;9:8421. doi: 10.1038/s41598-019-44815-x (PMC6557886; doi:10.1038/s41598-019-44815-x)
Supplement: Supplementary file 1 — Supplementary figures [file 41598_2019_44815_MOESM1_ESM.pdf]

The value of indocyanine green clearance assessment to predict postoperative liver dysfunction in patients undergoing liver resection

Christoph Schwarz[1], Immanuel Plass[1], Fabian Fitschek[1], Antonia Punzengruber[1], Martina Mittlböck[2], Stephanie Kampf[1], Ulrika Asenbaum[3], Patrick Starlinger[1], Stefan Stremitzer[1], Martin Bodingbauer[1], Klaus Kaczirek[1]

1 Department of Surgery, Division of General Surgery, Medical University Vienna

2 Section for Medical Statistics, Medical University Vienna

3 Department of Biomedical Imaging and Image Guided Therapy; Medical University Vienna

Supplementary figures

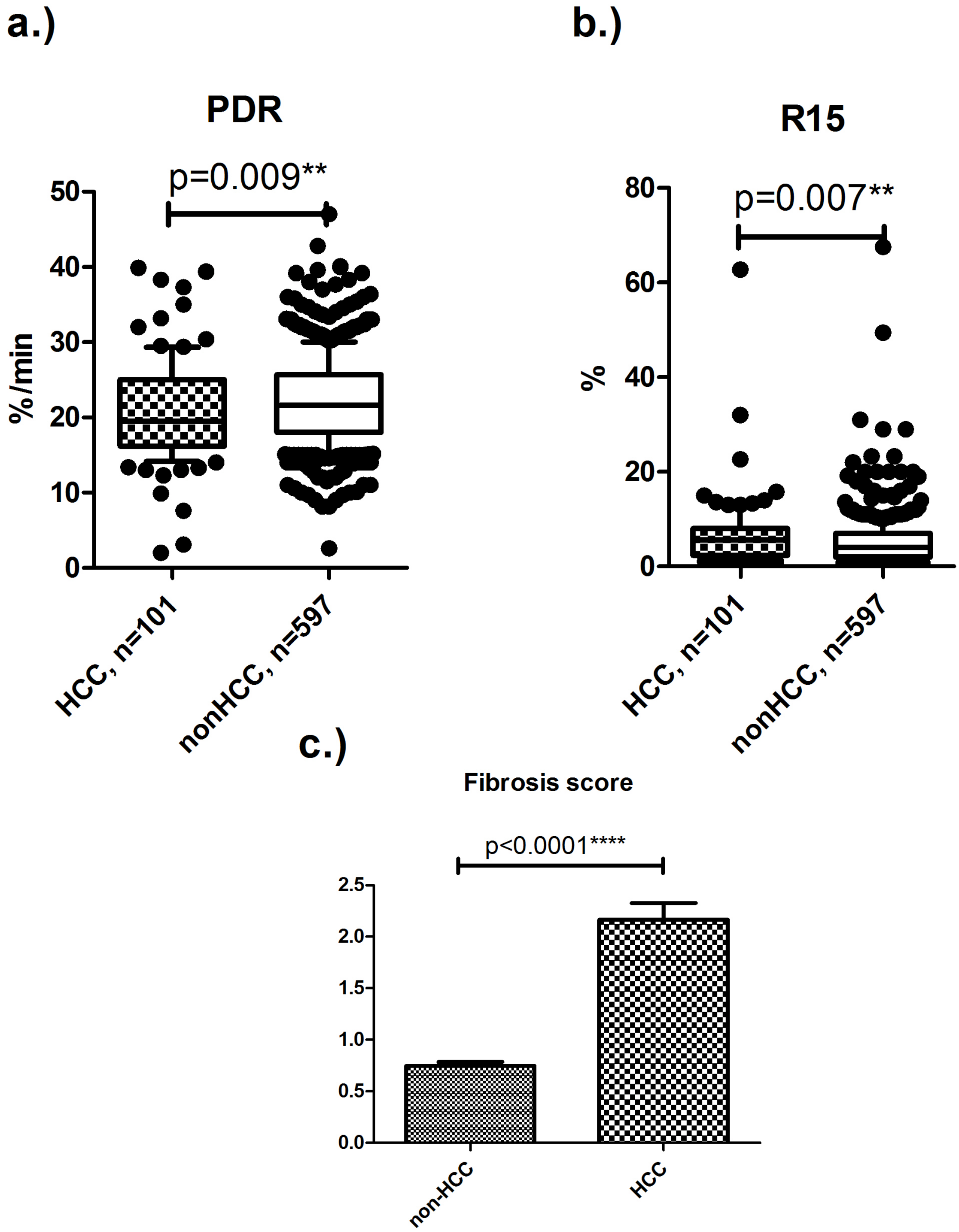

Suppl. Figure 1: HCC and ICG clearance: A, B) Patients with HCC had a significantly decreased ICG clearance compared to patients with another cause for liver resection. **C)** Notably, patients with HCC a significantly higher fibrosis score in the resected specimen. **p≤0.01; ****p<0.0001

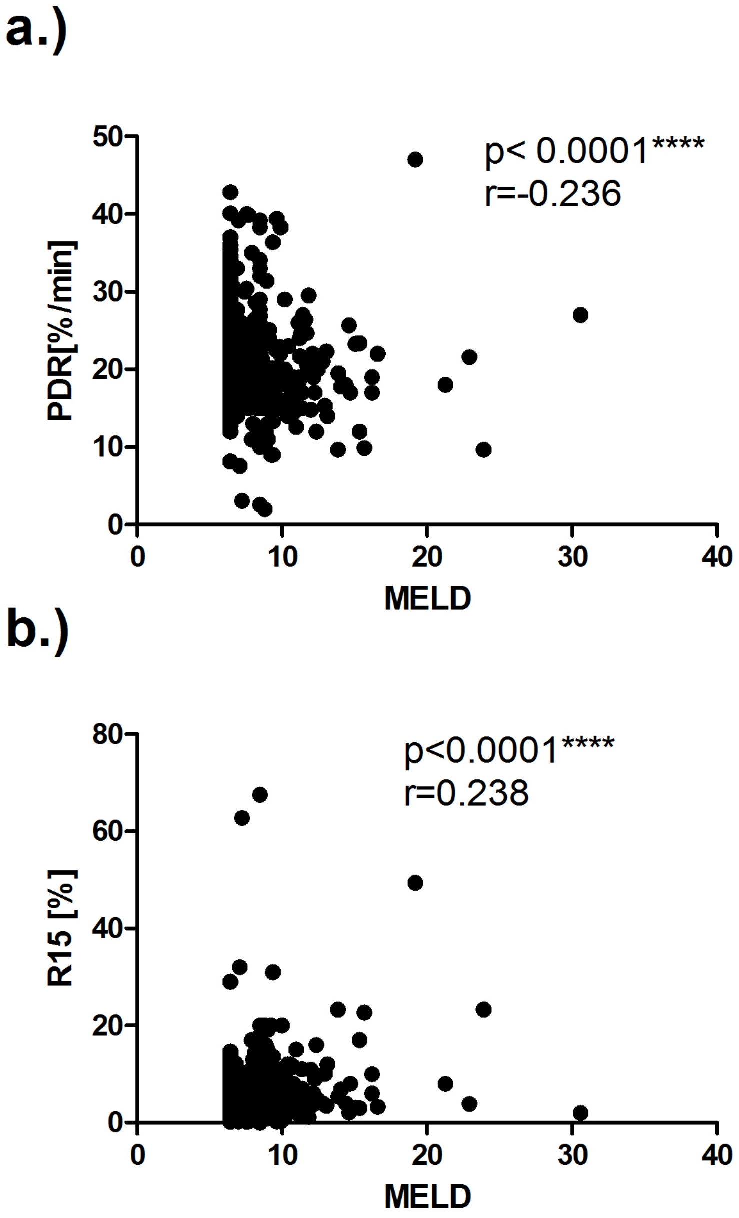

Suppl. Figure 2: MELD score and ICG clearance: There was a significant correlation between MELD score and ICG clearance expressed as PDR ($p<0.0001$; $r=-0.236$) and R15 ($p<0.0001$; $r=0.238$). ****$p<0.0001$
